# Philadelphia College of Pharmacy

**Published:** 1860-05

**Authors:** 


PHILADELPHIA COLLEGE OF PHARMACY.—The number of students in attendance at this old and flourishing institution during the last session was 125, being an increase of thirty-seven over the session of 1859–60. The degree of "Graduate in Pharmacy" was conferred upon twenty-nine gentlemen; and the valedictory address was delivered by Professor Thomas.

THE ARMY MEDICAL BOARD OF EXAMINERS, consisting of surgeons C. A. Finley, C. McDougall, and J. M. Cuyler, and assistant-surgeon J. F. Hammond, Recorder, will meet in the City of New York on the first of May, for the examination of assistant-surgeons for promotion, and of those candidates for appointment to the medical staff of the army as may have permission from the Secretary of War.

